# Mechanosensory encoding in ex vivo muscle–nerve preparations

**DOI:** 10.1113/EP090763

**Published:** 2023-04-29

**Authors:** Stephen N. Housley, Evelyn A. Gardolinski, Paul Nardelli, J'Ana Reed, Mark M. Rich, Timothy C. Cope

**Affiliations:** ^1^ School of Biological Sciences Georgia Institute of Technology Atlanta GA USA; ^2^ Department of Neuroscience, Cell Biology and Physiology Wright State University Dayton OH USA; ^3^ W.H. Coulter Department of Biomedical Engineering Emory University and Georgia Institute of Technology, Georgia Institute of Technology Atlanta GA USA

**Keywords:** electrophysiology, in vitro, mechanoreceptor, mouse, muscle spindle, proprioceptor, sensory, tendon organ

## Abstract

Our objective was to evaluate an ex vivo muscle–nerve preparation used to study mechanosensory signalling by low threshold mechanosensory receptors (LTMRs). Specifically, we aimed to assess how well the ex vivo preparation represents in vivo firing behaviours of the three major LTMR subtypes of muscle primary sensory afferents, namely type Ia and II muscle spindle (MS) afferents and type Ib tendon organ afferents. Using published procedures for ex vivo study of LTMRs in mouse hindlimb muscles, we replicated earlier reports on afferent firing in response to conventional stretch paradigms applied to non‐contracting, that is passive, muscle. Relative to in vivo studies, stretch‐evoked firing for confirmed MS afferents in the ex vivo preparation was markedly reduced in firing rate and deficient in encoding dynamic features of muscle stretch. These deficiencies precluded conventional means of discriminating type Ia and II afferents. Muscle afferents, including confirmed Ib afferents were often indistinguishable based on their similar firing responses to the same physiologically relevant stretch paradigms. These observations raise uncertainty about conclusions drawn from earlier ex vivo studies that either attribute findings to specific afferent types or suggest an absence of treatment effects on dynamic firing. However, we found that replacing the recording solution with bicarbonate buffer resulted in afferent firing rates and profiles more like those seen in vivo. Improving representation of the distinctive sensory encoding properties in ex vivo muscle–nerve preparations will promote accuracy in assigning molecular markers and mechanisms to heterogeneous types of muscle mechanosensory neurons.

## Abstract (highlights)

- **What is the central question of this study?**
  How well have studies using ex vivo  muscle‐ nerve preparations represented in vivo features of sensory encoding by low threshold mechanoreceptors in muscle?
- **What is the main finding and its importance?**
  We find experimental adjustments to the ex vivo approach that improve representation of mechanosensory encoding observed in vivo. These adjustments will enhance accuracy in the search for molecular identity and encoding mechanisms of heterogeneous muscle mechanosensory neurons.

## 1 INTRODUCTION

Mechanistic understanding of mechanosensory signalling remains fragmentary for primary sensory afferents supplying low threshold mechanoreceptors (LTMRs) in skeletal muscle. New hypotheses have developed, however, from recent discoveries of LTMR molecular composition and processes related to mechano‐transduction, spike train encoding and modulation of afferent excitability (Bewick & Banks, 2015; Blecher et al., 2018; Bewick & Banks, 2021; Oliver et al., 2021; Wu et al., 2021; de Nooij, 2022; Wilkinson, 2022). Advancing these proposals relies upon establishing relationships with afferent firing behaviours. Among available experimental approaches, the ex vivo muscle–nerve preparation developed by Wilkinson and colleagues (2012) presents advantages. By bypassing specialized equipment, experimental provisions, and training required by in vivo studies, the ex vivo preparation increases access to investigation of afferent firing behaviours. The ex vivo preparation also enables incisive tests of firing mechanisms using pharmacological agents or genetic mutations incompatible with in vivo study. Recent studies exploiting the ex vivo muscle–nerve preparation are generating novel mechanistic inferences about LTMR mechanosensory signalling in health and disease, for example the participation of Piezo 2, ASIC3, glutamate and acetylcholine receptors (Woo et al., 2015; Lin et al., 2016; Gerwin et al., 2019; Than et al., 2021; Espino et al., 2022; Bewick & Banks, 2021); the role of ion channels such as NaV_1.1_, (Woo et al., 2015; Gerwin et al., 2019; Than et al., 2021; Espino et al., 2022); and the effects of endotoxin‐induced inflammation (Zaytseva et al., 2018), muscular dystrophy (Gerwin et al., 2020) and joint disorders (Ma et al., 2023). Next steps in advancing these and anticipated new observations require critical examination of their distribution over the full functional range of specialized LTMR subtypes.

LTMR muscle afferents exhibit firing behaviours both shared and specialized in response to mechanical stimuli. All fire at increasing rates that slowly adapt as their parent muscle is stretched from one fixed position to another. LTMR afferents also differentially tune their firing to specific features of the muscle's mechanical responses (Matthews, 1972; Blum et al., 2020). Specialized tuning supports the conventional classification of LTMR muscle afferents into three stereotypical subtypes. Muscle spindle (MS) afferents separate into subtypes II and Ia, biased, respectively, in their encoding of muscle length and changes in length (Matthews, 1972; Banks et al., 2021; Vincent et al., 2017), while the third subtype, the tendon organ (TO, aka Ib) afferent tunes preferentially to active muscle force, but also responds to stretch of passive muscle (Jami, 1992; Matthews, 1972; Vincent et al., 2017). Variation in additional tuning properties, for example threshold, dynamic sensitivity and history dependence, also clusters around three primary afferent subtypes (Matthews, 1972; Vincent et al., 2017; Blum et al., 2020).

Similarities and differences in afferent encoding of muscle's mechanical responses depend upon the physical properties and mechanical environments of the receptors’ non‐neural elements (Schaafsma et al., 1991; Blum et al., 2020) together with the structural and biophysical properties of the receptors’ neuronal components (Matthews, 1972; Bewick & Banks, 2015; Housley et al., 2023). Afferent firing responses to muscle stretch measured in vivo reflect these dependencies, and deviation from these responses suggests underlying disorder(s). Such deviations appear in data obtained from an ex vivo muscle–nerve preparation extracted from wild‐type adult mice, wherein afferent encoding of muscle mechanical perturbations is suppressed and distorted relative to similar recordings obtained from healthy animals in vivo (Wilkinson et al., 2012; cf. Carrasco et al., 2017; Vincent et al., 2017). The present study was undertaken to perform a critical examination of LTMR firing properties obtained in the ex vivo muscle–nerve preparation.

## 2 METHODS

All procedures for the care and experimental use of animals in the present study were approved by the Georgia Institute of Technology Institutional Animal Care and Use Committee. A total of nine male and seven female wild‐type mice (strain C57BL/6 obtained from The Jackson Laboratory, Bar Harbor, ME, USA) ranging in age from 2 to 6 months and weight from 20 to 35 g were properly housed and subjected to no experimental treatment prior to their one‐time use in terminal experiments.

**FIGURE 1:**
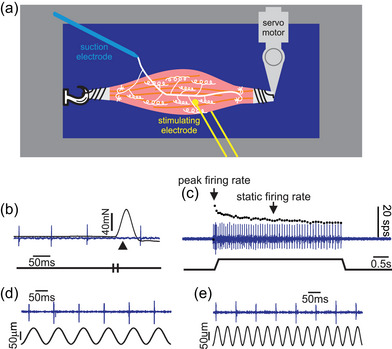
Recording paradigm and firing behaviours of a confirmed muscle spindle afferent. (a) Experimental features of ex vivo muscle–nerve preparation (see Methods for details). Muscle fixed and submersed in fluid‐filled chamber (HEPES‐buffer solution in this case). Suction electrode attached to nerve recorded spikes evoked by servo‐motor controlled stretch or muscle contraction elicited by bipolar stimulating electrode. Muscle spindle and tendon organ receptors (objects shown within the muscle) are indicated in locations taken from Sonner et al. (2017) for mouse soleus muscles. (b,c) Representative spiking activity (blue vertical lines) produced by one MS afferent in response to mechanical stimulation of EDL muscle at resting muscle length *L*_o_. (b) Electrical stimulation (bottom black trace indicating timing of two suprathreshold 50 μs pulses delivered at 100 spikes/s (sps)) delivered by stimulating electrode to induce isometric twitch (top black trace). Periodic spiking (blue vertical lines) produced by stretching muscle to >*L*_o_; arrowhead indicates expected time of occurrence of the next periodic spike that was instead delayed to the end of muscle twitch contraction (superimposed black trace). The afferent was classified MS by the pause in firing. Stimulus artifacts were removed from afferent firing record for clarity. (c) Ramp–hold–release muscle stretch (bottom trace) from *L*_o_ to 7.5% *L*_o_ at constant ramp and release velocities 60% *L*_o_/s temporally aligned with corresponding afferent spiking (blue middle trace) and instantaneous firing rate (IFR in spikes per second, sps; black dots), with peak and static firing rates defined. (d,e) Muscle length vibrations (bottom black traces) at 10 Hz (d) designated the maximum entrainment frequency by evoking a spike (top blue traces) with each vibration cycle in contrast with faster vibration frequencies, for example 25 Hz (e) that did not evoke cycle‐to‐cycle entrainment.

Terminal experiments approximated those described for studying the firing behaviours of LTMR afferents in ex vivo muscle–nerve preparations (Wilkinson et al., 2012). Each experiment began with a mouse deeply anaesthetized by isoflurane delivered first in an induction chamber followed by a nose cone over a period of 10–15 min concluding when both hindlimbs were surgically removed at the hip and the mice were killed by exsanguination. With the limbs bathed in dissecting solution (described below), the extensor digitorum longus (EDL) muscle in continuity with a short length (∼5 mm) of the severed common fibular nerve was carefully separated from other tissues and removed from the limb by cutting its tendons. The ex vivo muscle–nerve preparation was then transferred to a recording chamber (Figure 1a) where it was continually bathed in recording solution (described below) and secured by stretching the muscle between its tendon of origin tied to a rigidly fixed post and its tendon of insertion tied by 6‐0 suture directly to the rigid lever of a length‐servo motor (Aurora Scientific 300‐CLR, Aurora Scientific, Aurora, Ontario, Canada). By tying the tendon directly to the lever and leaving no length of suture interposed, we removed an artificial source of compliance which can alter muscle stretch and, in turn, affect afferent firing responses. The two ends of a bipolar silver electrode were placed on opposite sides of the muscle and used to evoke muscle contraction with suprathreshold current pulses (50 μs duration, 0.2 Hz). Resting muscle length (*L*_o_) was set at the optimal length for maximum twitch contraction force, and twitch contractions were evoked to test muscle health intermittently throughout data collection (≤4 h), which was discontinued when twitch force declined by ≥10%. Data were collected at either room temperature (24°C) or 34°C following Wilkinson et al. (2012).

An experimental variable central to the present study was the tissue bathing solution. In one case, we followed the protocol that Wilkinson and coworkers employed in their original (Wilkinson et al., 2012) and in several later reports (Zaytseva et al., 2018; Than et al., 2021; Espino et al., 2022; Ma et al., 2023). Upon removal from the animal, hindlimbs were submersed in a dissection solution continuously oxygenated (95% O_2_, 5% CO_2_) and consisting of (mM): 128 NaCl, 1.9 KCl, 1.2 KH_2_PO_4_, 26 NaHCO_3_, 0.85 CaCl_2_, 6.5 MgSO_4_, and 10 glucose (pH 7.4). The extracted muscle–nerve tissues were then transferred to a different solution used during data collection. That recording solution, here named HEPES‐buffer solution, was continuously oxygenated (100% O_2_) and contained (mM): 123 NaCl, 3.5 KCl, 0.7 MgSO_4_, 1.7 NaH_2_PO_4_, 2.0 CaCl_2_, 9.5 NaC_6_H_11_O (sodium gluconate), 5.5 glucose, 7.5 sucrose, and 10 HEPES (pH 7.4). In separate experiments, extracted tissues were bathed during both dissection and recording in a continuously oxygenated (95% O_2_, 5% CO_2_) solution named Bicarb‐buffer solution (Nakanishi et al., 2005), which consisted of (mM): 118 NaCl, 3.5 KCl, 26.2 NaHCO_3_, 2 CaCl_2_, 0.7 MgSO_4_, 5.5 glucose, and 1.7 NaH_2_PO_4_ (titrated to pH 7.4 with NaOH).

Procedures for data collection and analysis approximated those used by Wilkinson et al. (2012). Briefly described, the open tip end of a glass micropipette (diameter 10–50 μm) containing the recording bath solution was attached by suction to a fraction of the nerve's cut surface. Recording started when muscle stretch evoked visually discriminable extracellular action potentials, that is, spikes. Under those conditions, the afferents were first classified by their response to isometric twitch contractions evoked by electrical stimulation applied through bipolar electrode wires placed directly on the muscle. Afferents producing spikes elicited during the rising phase of twitch force were designated TO afferents. Some TO afferents spiked only when the force of contraction was increased by temporal summation of successive twitches evoked by paired electrical stimuli temporally spaced at a short interval (10 ms). Afferents that paused firing during the twitch were designated MS afferents (Figure 1b). In some cases, it was necessary to stretch the muscle to lengths >*L*_o_ to produce the steady afferent firing required to observe a pause caused by muscle contraction. Next, and with the muscle set at resting length *L*_o_ (systematically varied in some instances), the servomotor under computer control generated a battery of stretch paradigms, including ramp–hold–release stretch (amplitude 2.5%, 5.0% and 7.5% *L*_o_; ramp and release rates 20%, 40% and 60% *L*_o_/s); muscle vibration at four different frequencies (10, 25, 50 and 100 Hz) and four different amplitudes (5, 25, 50 and 100 μm). These stretch paradigms were presented sequentially, each one repeated in multiple trials.

All extracellular records of spiking activity together with records of muscle force and length were digitized (20 kHz), streamed and stored in files (Power1401 and Spike2 software; Cambridge Electronic Design, Cambridge, UK) on computer for offline analysis. Muscle afferents were discriminated and analysed offline using, respectively, the Spike2 template spike sorting module and a custom analysis script. The analysis script extracted measured parameters, including instantaneous firing rates in spikes/s (sps) for peak dynamic firing at ramp peak and static firing at hold‐phase mid‐point (Figure 1c). Dynamic index was calculated by subtracting static from dynamic instantaneous firing rates. Visual inspection determined the highest frequency of muscle vibration, up to 100 Hz, that entrained afferent spikes one for one with each cycle of vibration (Figure 1d,e).

## 3 RESULTS

The present study aimed to test the ability to differentiate LTMR muscle afferents by their firing behaviours in ex vivo preparations. Firing of individual neurons responding to muscle stretch or contraction was discriminated among action potentials recorded extracellularly from the axons in the peripheral nerve innervating EDL muscles. Data were collected from our close reproduction of the ex vivo preparation developed and propagated by Wilkinson et al. (2012) and Wilkinson (2022). Data sets were generated in separate experiments using either the HEPES‐buffer or Bicarb‐buffer recording solutions (see Methods). No statistically significant differences were detected in afferent firing behaviours between male and female mice (ANOVA, Tukey *post hoc*, *P* > 0.05).

### 3.1 Firing properties of confirmed MS afferents in HEPES‐buffer solution

**FIGURE 2:**
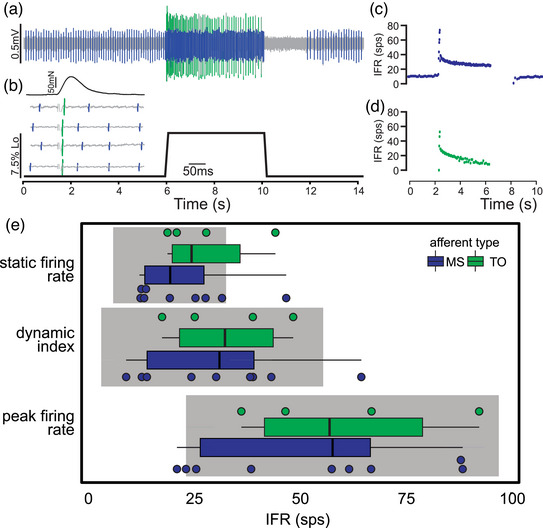
Ambiguity in distinguishing spike encoding of muscle stretch between MS and TO afferents in HEPES‐buffer solution. (a) Representative case of spiking activity recorded simultaneously from one muscle spindle (MS: blue) and one tendon organ (TO: green) afferent in response to ramp–hold–release stretch (bottom trace). Inset (b) shows four trials of spiking during isometric twitch contraction that consistently interrupted or initiated spiking, respectively, to confirm MS and TO afferent identity. Note that pauses in slow background firing rates for the MS afferent were sometimes difficult to identify and required multiple trials for confirmation. (c,d) Coloured circles plot instantaneous firing rates generated by the MS (c) and TO (d) afferents in response to ramp–hold–release stretch. (e) Encoding parameters: static firing rate, dynamic index and peak firing rate, for individual afferents (filled circles) averaged from four trials of ramp–hold–release (7.5% *L*_o_ at constant velocity 60% *L*_o_/s) plotted for all colour‐coded TO and MS afferents together with corresponding box and whisker plots (markers for median, quartiles and range). Grey rectangles document the minimum and maximal values estimated from data reported in Wilkinson et al. (2012) to show similarity of firing responses recorded under identical conditions in this study.

Figures 1 and 2 show representative firing behaviours of afferents with MS and TO identities confirmed by firing responses to isometric twitch contractions (Figures 1b and 2b). For both afferent subtypes, ramp–hold–release muscle stretch evoked a slowly adapting firing pattern characteristic of all LTMR muscle afferents: firing rate rose to a peak at the end of ramp stretch then slowly adapted as it fired throughout the stretch hold phase (Figure 1c; Figure 2c,d). Figure 2e plots standard measures of firing recorded in HEPES‐buffer solution in response to ramp–hold–release muscle stretch for all nine confirmed MS afferents. (Data taken from a fuller range of stretch rates and amplitudes are reported in Supplemental findings). Firing properties in our relatively small sample of MS afferents aligned well with properties of the 53 afferents (9 of which were experimentally confirmed MS) reported by Wilkinson et al. (2012) under corresponding experimental conditions at 24°C. For 8/9 MS afferents in our sample, values for static and dynamic firing and for dynamic index fell within or close to the range established by Wilkinson and coworkers (2012) (Figure 2e).

Our results also replicated those of Wilkinson et al. (2012) in demonstrating conspicuous differences in afferent firing behaviours measured with HEPES‐buffer solution versus in vivo (cf. Carrasco et al. (2017) for mouse Ia afferents; Vincent et al. (2017) for rat MS afferents). Absolute values for peak and static firing rates were markedly lower than those recorded in vivo. For example, static and peak firing rates, respectively, were ca. 45 spikes/s and 190 spikes/s slower on average than those recorded in vivo from mouse MS subtype Ia afferents. In addition, none of the MS afferents sampled in HEPES‐buffer solution exhibited initial burst firing (cf. inset in Figure 4a,b), and only 1/9 entrained firing with muscle vibration at frequencies greater than 25 Hz (e.g. Figure 1d,e). Partial or complete suppression of these dynamic firing responses eliminated criteria used by in vivo studies to differentiate Ia from II MS subtypes, for example Matthews (1972) and Vincent et al. (2017). These exclusions would also limit comprehensive assessment of treatment effects on the dynamic firing properties of MS afferents.

### 3.2 Ambiguity in distinguishing confirmed MS and TO afferents in HEPES‐buffer solution

We next asked whether TO afferents fired in response to stretch of non‐contracting muscle as they do in rat in vivo (Vincent et al., 2017), and if so, were they were *indistinguishable* from MS afferents. Figure 2a provided affirmative answers to both questions as illustrated by a case in which the spikes of one afferent of each type were discriminable in the same recording. First, the TO afferent, confirmed by spiking during the rising phase of twitch contraction (Figure 2b), readily fired in response to ramp–hold–release stretch as did the confirmed MS afferent under identical stretch conditions (Figure 2a). Second, the firing profiles evoked by ramp–hold–release stretch were roughly similar for the two afferents (Figure 2c,d). The absence of repetitive firing of TO afferents at resting muscle length *L*_o_ (Figure 2a) was not sufficient to distinguish them from MS afferents, since we observed no firing at *L*_o_ in 5/9 MS afferents sampled (e.g. Figure 1c). Therefore, repetitive firing at resting muscle length *L*_o_ was not sufficient to differentiate TO from MS afferents contrary to earlier assertions (Franco et al., 2014).

Our pooled sample represented both MS and TO afferents consistent with the known presence of both receptor types in the mouse EDL (Soukup, 1983; Stephens, 1985). Figure 2e shows that standard measures of firing to ramp–hold–release stretch for MS and TO afferent types were distributed over nearly co‐extensive ranges. Figure 2e also shows that the firing properties of TO afferents fell within the range reported by Wilkinson et al. (2012) for afferents designated, yet inconsistently confirmed as MS, thereby supporting the likely possibility that their sample unknowingly included TO afferents.

For 2/4 confirmed TO afferents, we tested firing responses to ramp–hold–release stretch superimposed on different resting muscle lengths (see Supplemental findings). Both TO afferents fired in response to ramp–hold–release stretche amplitudes of ≤6% *L*_o_ with the characteristic profile shown in Figure 2d at *L*_o_, and one fired at ½ *L*_o_, although at reduced firing rates. Firing by the other TO afferent fell below threshold when background stretch was reduced to ½ *L*_o_, consistent with slightly higher stretch thresholds for TO versus MS afferents (Vincent et al., 2017). These findings fit with overlapping distributions of stretch thresholds observed for TO and MS afferents in vivo (Vincent et al., 2017), and they refute assertions that the firing thresholds of TO afferents are uniformly outside the physiological range of passive muscle stretch.

**FIGURE 3:**
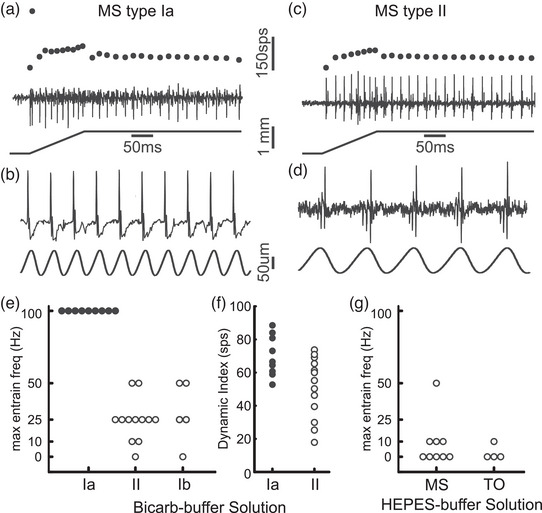
Muscle spindle subtypes classified by conventional measures in Bicarb‐buffer solution. Firing patterns observed in two confirmed MS afferents. (a,c) Simultaneous records of ramp–hold changes in muscle length (bottom length traces), evoked spikes (middle traces) and instantaneous firing rates (top traces). Presence versus absence of high frequency initial burst firing at stretch onset, respectively, distinguish type Ia versus II afferents (see also Figure 4a, b). (b,d) Simultaneous records of muscle length during vibration (bottom traces) and evoked spikes at maximum entrainment frequency (top traces). (b) High (100 Hz) versus low (25 Hz) frequency entrainment, respectively, typify type Ia versus II afferents. (e) Values of maximum entrainment frequency plotted for each afferent (circles) sampled in Bicarb‐buffer solution at 24°C; filled versus open circles, respectively, represent presence or absence of initial burst firing determined during ramp–hold–release stretch (data not shown). High frequency entrainment coupled with initial burst designated subtype Ia afferents; the converse of these two properties designated subtype II afferents. (f) dynamic index for afferents represented by circles as described in (e). Shift toward higher values of dynamic index expected for subtype Ia versus II afferents. (g) Plot same as (e) for a separate set of afferents sampled in HEPES‐buffer solution.

None of the firing parameters encoding various features of passive muscle stretch studied here reliably differentiated confirmed TO from MS afferent types in HEPES‐buffer solution. As another example, the response to muscle vibration for some TO afferents matched the maximum vibration frequency evoking one to one firing entrainment exhibited by MS afferents (see Figure 3g).

The present findings establish ambiguity in distinguishing *individual* TO from MS afferents by any physiological measure other than the twitch test in the ex vivo preparation with HEPES‐buffer solution. Absent confirmation by the twitch test, TO afferents would have been masked in our pooled sample. Furthermore, suppression of dynamic firing properties examined here prevented unequivocal discrimination of MS subtypes Ia and II. In sum, none of the LTMR muscle afferent subtypes could be identified with certainty in the HEPES‐buffer solution based solely on afferent firing responses to passive muscle stretch.

### 3.3 Firing properties of afferent subtypes in Bicarb‐buffer solution

In separate experiments, we examined afferent firing in the same ex vivo muscle–nerve preparation using a different bathing solution (see Methods). Bicarb‐buffer solution was adopted from our earlier study of neuromuscular junction physiology in an ex vivo preparation of skeletal muscle (Nakanishi et al., 2005). That Bicarb‐buffer solution approximates ion concentrations, pH and osmolarity found in normal interstitial fluid (Burgess & Sylven, 1962; Fogh‐Andersen et al., 1995; Rich et al., 1998), and it excludes sodium gluconate and sucrose present in the HEPES‐buffer solution.

**FIGURE 4:**
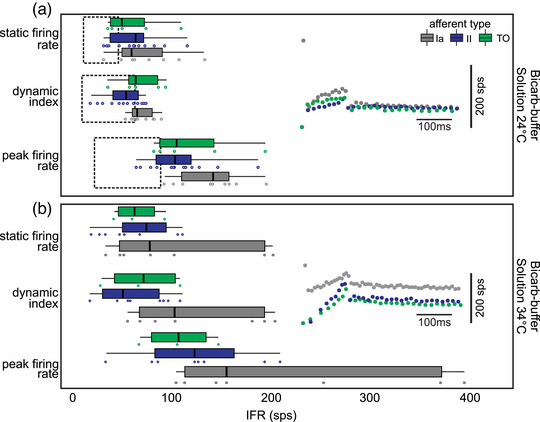
Spike encoding properties of the three main muscle LTMR subtypes in Bicarb‐buffer solution. Spike encoding parameters (static firing average, dynamic index and peak firing rate) measured ex vivo for two separate afferent samples studied in Bicarb‐buffer solution at 24°C (a) or 34°C (b). Values for individual afferents plotted as circles together with corresponding box and whisker plots (markers for median, quartiles and range). Overlain traces to the right side of each panel show instantaneous firing rate profiles generated by single afferents of colour‐coded identity in response to ramp–hold–release stretch ( 7.5% *L*_o_ at constant velocity 60% *L*_o_/s). Note the high frequency initial burst firing at stretch onset for type Ia afferents. For comparison, dashed‐line rectangular boxes in (a) outline ranges for various firing properties found for MS and TO afferents sampled in the present study in HEPES‐buffer solution at 24°C (Figure 2).

Several features of afferent firing in the Bicarb‐buffer solution exhibited clear differences from those in HEPES‐buffer solution. Perhaps most important was the emergence of unique dynamic firing properties that enabled standard means of discriminating MS subtypes. Initial burst firing at rates exceeding 100 spikes/s appeared at the onset of rapid muscle stretch (>40% *L*_o_/s) in a subset of confirmed MS afferents (e.g., Figures 3, 4). Nine of those 10 afferents also fired spikes with every cycle of muscle vibration at 100 Hz (Figure 3b). Specified values for initial burst firing and entrainment to high frequency vibration (Figure 3b) are each unique identifiers of MS subtype Ia afferents(Brown et al., 1967; Hunt & Ottoson, 1976; Scott, 1992; De‐Doncker et al., 2003). Here as in our earlier in vivo studies (Carrasco et al., 2017; Vincent et al., 2017; Blum et al., 2020), we restricted the subtype Ia designation to afferents that co‐expressed those two properties. Note that none of the nine MS afferents studied in HEPES‐buffer solution met either criterion (Figure 3g). In the Bicarb‐buffer solution we also sampled 13 confirmed MS afferents that were named subtype II for expressing neither initial bursts nor high frequency vibration entrainment (Figure 3c–e). Three MS afferents were not classifiable by these standards and were excluded from further study. Consistent with our MS subtype classification, values for dynamic index skewed toward higher values for Ia than for II subtypes as long established (Figure 3f; Matthews, 1972).

Figure 4 shows standard measures of firing recorded in response to ramp–hold–release muscle stretch for LTMR muscle afferents classified into three main subtypes using criteria described above (data taken from a fuller range of stretch rates and amplitudes are reported in Supplemental findings). Figure 4a illustrates that firing properties of afferents sampled in the Bicarb‐buffer solution expanded to higher values relative to distributions established for afferents in the HEPES‐buffer solution. Values for static and peak dynamic firing rates and dynamic index shifted toward even higher values when the Bicarb‐buffer solution was warmed from 24°C to 34°C for a separate sample of classified afferents (Figure 4b). At the warmer temperature, which is within 2.5°C of in vivo muscle temperature (Sharma et al., 2021), firing properties for subtype Ia extended values into the range established for the Ia afferents recorded from mice in vivo (cf. Carrasco et al., 2017). Comparisons cannot be made for mouse subtypes II and Ib for which no in vivo data are available.

## 4 DISCUSSION

Our objective was to compare and contrast firing behaviours of LTMR subtypes in an ex vivo muscle–nerve preparation developed and propagated by Wilkinson and colleagues. In our replication of that preparation, firing responses to passive muscle stretch were insufficient to achieve unambiguous classification of afferent subtypes. By showing that samples of MS and TO afferents generated overlapping firing rates and patterns in response to the same parameters of muscle stretch, our results refute assumptions that stretch‐evoked firing in the ex vivo preparation is solely attributable to MS afferents. For MS afferents, firing rates and conventional measures of dynamic firing responding to muscle length changes were blunted to the point that type Ia and II afferent were indistinguishable. However, switching the recording solution from a HEPES to a bicarbonate buffer restored dynamic firing behaviours conventionally used to discriminate MS subtypes Ia and II in vivo. We conclude that experimental modifications like those demonstrated here are needed to enable use of the ex vivo muscle–nerve preparation to accurately map molecular identity and mechanisms onto the three main muscle LTRM subtypes.

### 4.1 Relevance to earlier studies

The present findings substantiate uncertainty about afferent identity in published reports using an ex vivo muscle–nerve preparation. First, our findings suggest the likely, yet unrecognized, inclusion of TO afferents. Afferents sampled in the present study using HEPES‐buffer solution included confirmed TO afferents, most of which responded to passive muscle stretch with firing patterns indistinguishable from MS afferents (Figure 2). Stretch sensitivity of TO afferents was not surprising to us given earlier, though conflicting results in studies of cat (Jami, 1992). Our studies of rat afferents in vivo (Bullinger et al., 2011; Vincent et al., 2017; Housley et al., 2021; Housley et al., 2022) consistently demonstrated that the majority of TO afferents fire readily in response to magnitudes of muscle stretch similar to or only slightly greater than for subtype II MS afferents. Based on the ratio of ca. 2:1 MS:TO afferents found in our relatively small sample, we expect that TO afferents were mistaken for MS afferents in earlier studies, in which the type‐definitive twitch contraction test was applied inconsistently (Wilkinson et al., 2012) or not at all (Than et al., 2021; Espino et al., 2022; Wilkinson, 2022). Second, nearly all afferents sampled in HEPES‐buffer solution expressed a MS subtype II phenotype, that is, they entrained firing only to low muscle vibration frequencies (generally <25 Hz), they lacked substantial initial burst firing, and they expressed relatively low values for dynamic index. Given anatomical proof of their presence in mouse hindlimb muscle (Sonner et al., 2017), group II afferents are likely included when sampling among undifferentiated MS afferents contrary to earlier suggestions (Wilkinson et al., 2012; Gerwin et al., 2019). However, subtype Ia afferents are prevalent among mouse LTMRs (Carrasco et al., 2017; Sonner et al., 2017) and were also likely present in random samples of MS units in the ex vivo preparation with HEPES‐buffer solution just as they were in Bicarb‐buffer solution. Although probably sampled in earlier studies, Ia afferents could not be confirmed because HEPES‐buffer solution blunted their distinctive firing properties (see below). All considered, none of the three primary LTMR muscle afferent subtypes could be identified with certainty in cases relying solely on afferent firing responses to passive muscle stretch in the HEPES‐buffer solution.

Dynamic firing behaviour expected among confirmed MS afferents was conspicuously absent in HEPES‐buffer solution. No initial bursts were observed and afferents rarely entrained firing to muscle vibration ≥10 Hz (Figure 2). Values of dynamic firing rates and dynamic index were nearly co‐extensive for confirmed MS and TO afferents. Suppressed encoding confounds mechanistic inferences taken from the effects of experimental perturbations on dynamic firing. For example, recent reports demonstrate that reducing expression of the ion channel NaV1.1 or blocking glutamate release impairs static but not dynamic firing properties of muscle spindle afferents (Than et al., 2021; Espino et al., 2022). However, blunting dynamic firing properties would be expected to obscure treatment effects on dynamic encoding. These examples support our conclusion that the ex vivo muscle–nerve preparation in HEPES‐buffer solution is limited in evaluating some features of mechanosensory encoding and in assessing specializations in mechanosensory encoding behaviours of LTRM muscle subtypes.

### 4.2 Recording solutions

Our study was not primarily intended to perform an investigation of how the different compositions of recording solutions affect the physiology of the ex vivo muscle preparation. Nonetheless, ample knowledge of the physiological role of bicarbonate in regulating nerve and muscle excitability, for example Jones et al. (2014), draws attention to bicarbonate's exclusion from the HEPES‐buffer solution. For example, the omission of HCO_3_^−^ and CO_2_ from the extracellular medium altered membrane potential and ion conductance in an in vitro study of hippocampal pyramidal neurons (Church, 1992). Evidence suggests that changing bicarbonate acts on neuronal excitability through the pH dependency of ion channel gating for Na^+^, Ca^2+^, and K^+^ channels (Nonner et al., 1980; Davies et al., 1992; Imber et al., 2014). Modulation of ion channels seems a likely explanation for suppression of dynamic firing observed caused by raising pH in the extracellular medium bathing isolated muscle spindles in vitro (Fischer & Schäfer, 2005). Bicarbonate also regulates chloride concentration, and therefore Cl^−^ equilibrium potential, in part via Cl^−^–bicarbonate exchangers such as SLC4A2 (Grossie, 1985; Terry et al., 2018). The variety of ion channels resident within muscle spindles (Bewick & Banks, 2015; Carrasco et al., 2017; Housley et al., 2023) presents a complex set of sites and processes by which altering bicarbonate homeostasis might disturb electrical signalling involved in mechanotransduction and spike encoding. Independent of its effects on the spindle's neural components, changing proton buffering might also contribute to afferent firing abnormalities by altering pH exposure of non‐neural components and processes, for example cross‐bridge cycling of intrafusal muscle fibres (Westerblad et al., 1997).

While it is difficult to predict the aggregate effect of bicarbonate exclusion on afferent firing, that is, whether suppression or amplification, other reports join ours in supporting net suppression. The exclusion of bicarbonate buffer in an ex vivo skin–nerve preparation yields markedly reduced firing by LTRM cutaneous afferents compared to that observed in vivo (Koltzenburg et al., 1997; Housley et al., 2021, 2022). Conversely, the inclusion of bicarbonate employed in some studies of the same ex vivo preparation studied here substantially relieved firing suppression in stretch sensitive afferents (de Nooij et al., 2015; Lin et al., 2016; Gerwin et al., 2019, 2020). Other factors contributing to the deviation from in vivo firing in HEPES‐buffer solution may include the addition of gluconate and sucrose in recording solutions. Nonetheless, our Bicarb‐buffer solution did not comprehensively preserve the firing rates and patterns of afferents observed in vivo, and not surprisingly so given the unphysiological conditions inevitably introduced by extracting nerve and muscle from the animal.

### 4.3 Recommended classification protocols

Present findings together with observations in earlier reports lead us to offer the following recommendations for improving confidence and specificity in classifying LTMR muscle afferent subtypes in the ex vivo muscle–nerve preparation developed by Wilkinson et al. (2012). At the outset, we caution that subtype designation by our recommended criteria is provisional since firing behaviours are only a proxy for definitive classification by distinctions in muscle receptor innervation. Nonetheless, the criteria we recommend here for afferent subtype classification in the ex vivo muscle–nerve preparation gain credibility from their conventional application in numerous in vivo studies (Matthews, 1972). First, we emphasize that reliable identification of MS and TO afferents requires testing each one for its firing response to isometric twitch contractions of the parent muscle. Second, the Bicarb‐buffer solution enables discrimination of MS subtypes Ia versus II by measures of dynamic firing, namely initial burst firing and firing entrainment to high frequency (≥100 spikes/s) muscle vibration, both of which are best revealed by rapid rates (≥60% *L*_o_/s) of muscle stretch. Third, we corroborate the increase in firing responsiveness obtained by warming the recording solution to the physiological temperature range, ca. 34°C, which in combination with the Bicarb‐buffer solution moves measures of stretch‐evoked firing closer to the range observed for muscle afferents in vivo. While some differences from in vivo afferent firing behaviour persist, notably aspects of dynamic firing, our recommendations for the ex vivo muscle–nerve preparation reveal the heterogeneous subtypes of LTMR as needed to accurately assign associated molecular markers and mechanisms.

## AUTHOR CONTRIBUTIONS

Conception or design on work: Timothy C. Cope, Stephen N. Housley, Paul Nardelli, Mark M. Rich. Acquisition, analysis, or interpretation of data for the work: Evelyn A. Gardolinski, Paul Nardelli, J'Ana Reed, Mark M. Rich, Stephen N. Housley, Timothy C. Cope. Drafting of the work or revising it critically for important intellectual content: Timothy C. Cope, Stephen N. Housley, Evelyn A. Gardolinski, Paul Nardelli, J'Ana Reed, Mark M. Rich. All authors have read and approved the final version of this manuscript and agree to be accountable for all aspects of the work in ensuring that questions related to the accuracy or integrity of any part of the work are appropriately investigated and resolved. All persons designated as authors qualify for authorship, and all those who qualify for authorship are listed.

## CONFLICT OF INTEREST

The authors declare that they have no known competing financial interests or personal relationships that could have appeared to influence the work reported in this paper.

## Supporting information

Supplemental findings
